# Association between lifestyle factors and the risk of metabolic syndrome in the South Korea

**DOI:** 10.1038/s41598-022-17361-2

**Published:** 2022-08-03

**Authors:** Yu Shin Park, Soo Hyun Kang, Sung-In Jang, Eun-Cheol Park

**Affiliations:** 1 Department of Public Health, Graduate School, Yonsei University, Seoul, Republic of Korea; 2 Institute of Health Services Research, Yonsei University, Seoul, Republic of Korea; 3 Department of Preventive Medicine, Institute of Health Services Research, Yonsei University College of Medicine, 50 Yonsei-to, Seodaemun-gu, Seoul, 03722 Republic of Korea

**Keywords:** Health care, Risk factors

## Abstract

This study aimed to examine the association between lifestyle factors and metabolic syndrome risk in South Korean adults. Korea National Health and Nutrition Examination Survey 2016–2018 data were used. The study included 6,995 subjects (2835 male; 4,160 female). Multiple logistic regression was used to estimate the relationship between the lifestyle factors, including sedentary time, sleep duration, alcohol consumption, smoking status, and dietary intake. Metabolic syndrome prevalence in healthy adults was 25.6% and 12.4% in men and women, respectively. Male with over four lifestyle risk factors had a higher OR for metabolic syndrome risk (over four lifestyle factors: OR 1.97, CI 1.18–3.27). Female with more than one lifestyle risk factor had a higher OR for metabolic syndrome risk (one lifestyle factor: OR 1.58, CI 1.10–2.28; two lifestyle factors: OR 2.08, CI 1.39–3.11; three lifestyle factors: OR 1.94, CI 1.20–3.13). In particular, female with more lifestyle factors had increased likelihood of abdominal obesity, hypertension, and high triglycerides. Male with more lifestyle factors had increased likelihood of high triglycerides. Sedentary time was significantly associated with increased metabolic syndrome in male and female. This study found a significant association between the number of lifestyle risk factors and the risk of metabolic syndrome in Korean adults. The greater the number of lifestyle risk factors, the higher the risk of metabolic syndrome in both sexes. People with a greater number of poor lifestyle behaviors tended to exhibit increased likelihood of especially elevated triglyceride levels.

## Introduction

Due to the association between cardiovascular disease and metabolic syndrome as well as their common occurrence, researchers have exhibited keen interest in these conditions^1^. People with metabolic syndrome have high risks of morbidity and mortality because of cardiovascular disease and type 2 diabetes^2^. The prevalence of metabolic syndrome in Korean adults increased from 24.9% in 1998 to 31.3% in 2007. In other words, approximately one in three adults in Korea has metabolic syndrome^3^. Further, cardiovascular disease incidence has continuously increased over the past 10 years and has become the second most common cause of mortality in Korea^4^. Due to increasing disease duration and accompanying disabilities, the socioeconomic burden posed by these diseases is predicted to increase^5^. Therefore, it is important to prevent and reduce the prevalence of metabolic syndrome.

The World Health Organization (WHO) defined metabolic syndrome as a pathologic condition comprising abdominal obesity, insulin resistance, hypertension, and hyperlipidemia. Metabolic syndrome is not a single disease but a group of risk factors for cardiovascular disease^6^.

The main strategy for metabolic syndrome prevention and treatment are the change of poor lifestyle through various approach based on physical exercise, a healthy diet and education. It means that risk factors for metabolic syndrome can be mitigated by modifiable lifestyle factors^7^. To support this opinion, there are various studies related to risk factor of metabolic syndrome and lifestyle intervention for metabolic syndrome^8,9^. For instance, prior studies suggests that lifestyle risk factors, such as poor diet, alcohol consumption, physical inactivity, smoking, and sedentary time increase the risk of metabolic syndrome^9–12^.

According to recent studies, sleep duration is also a lifestyle factor that potentially acts as an important health status indicator^13^. To reduce the prevalence of metabolic syndrome, lifestyle and pharmacological method modifications are of paramount importance.

Whereas many studies are focused on the prevalence of single- or double-risk factors and their association with metabolic syndrome, including that in people with comorbid disease^14,15^, we investigated the association of overalllifestyle factors with metabolic syndrome and its affected components of metabolic syndrome, excluding that in patients with comorbid disease. Such a design potentially demonstrates the prevalence, trends, and determinants of metabolic syndrome. Accordingly, in the present study, we used cross-sectional data to examine the association of lifestyle risk factors with the risk of metabolic syndrome and its components. An enhanced understanding of the association between lifestyle behavior and metabolic syndrome potentially improves the planning of new effective health programs and result in improved health outcomes.

## Methods

This cross-sectional study used data from the 2016–2018 Korea National Health and Nutrition Examination Survey (KNHANES) conducted by the Korea Centers for Disease Control and Prevention. The KNHANES is a cross-sectional, nationally representative survey that has been conducted regularly since 1998 to examine the general health and nutritional status of Korean citizens. Our study did not require Institutional Review Board approval because the KNHANES provides a secondary dataset, which is available in the public domain and does not contain private information. And respondents consented the survey for collecting data before participating in the survey^16^.

### Participants

The total survey population from 2016 to 2018 included 24,269 individuals. Individuals younger than 19 years of age (n = 4880) were excluded. Moreover, we excluded individuals undergoing treatment for previously diagnosed hypertension (n = 4223), diabetes mellitus (n = 1697), and hyperlipidemia (n = 2358) as well as those with diagnoses of angina (n = 362), myocardial infarction (n = 209), and stroke (n = 421). Finally, 6995 participants were selected for participation in this study after excluding missing data (n = 7,347).

### Variables

The main variables in this study were the number of abnormal lifestyle among the following five lifestyle factors: sleep duration, sedentary time, alcohol use disorders, smoking status, and dietary intake, the data of which were self-reported. Sleep duration was assessed using the following question: “How long do you usually sleep on a weekday and weekend?” Sleep duration was calculated as the total five-weekday plus total two-day-weekend sleep durations divided by 7 days. Subjects were categorized into two groups: (1) short sleep (< 6 h) and long sleep (≥ 9 h) duration and (2) normal sleep duration (6 h ≤ and < 9 h). Sedentary time was assessed using the following question: “How much time do you usually spend sitting?” The sedentary time was then calculated by dividing the sitting time by the time spent awake. Thereafter, we set the median as the cutoff, resulting in one category being Q1–Q2 and the other Q3–Q4: (1) normal sedentary time (Q1–Q2) (2) long sedentary time (Q3–Q4). In a previous study, shorter sleep duration was found to be related to a longer sedentary time^17^. We used the Alcohol Use Disorders Identification Test (AUDIT-C) score to assess the pattern of alcohol use disorders as follows:

1. “How often have you had a drink containing alcohol in the last year?”
2. “How many drinks containing alcohol did you have on a typical drinking day in the last year?”
3. “How often in the last year have you had six or more drinks on one occasion?”

A high-risk alcohol level was assigned an AUDIT-C score ≥ 8 for both sexes. Subjects were categorized into two groups: (1) never-mild drinking (AUDIT-C < 8), (2) heavy drinking (AUDIT-C ≥ 8). Smoking status was divided into the following two categories: (1) ex- or never-smoker and (2) current smoker. Dietary intake was assessed using the 24-h dietary recall method. We categorized carbohydrate and fat consumption reflected current dietary by cutoff which was recommended by the Korea Ministry of Health and Welfare and the Korean Nutrition Society^18,19^: poor dietary pattern was defined as having one or two of the following two components: (1) high fat intake corresponded to exceeding 30% of dietary energy intake and (2) high carbohydrate intake corresponded to exceeding 65% of dietary energy intake. If either component is applicable, it was assigned to the abnormal group.

The dependent variable was metabolic syndrome. The definition provided by the modified Third National Cholesterol Education Program Expert Panel on Detection, Evaluation, and Treatment of High Blood Cholesterol in Adults as well as the specific waist circumference values provided by the WHO and Korean Society for the Study of Obesity were used to determine metabolic syndrome and its components^20^, which were as follows: (1) abdominal obesity (waist circumference ≥ 90 cm in men and ≥ 85 cm in women), (2) high blood pressure (systolic ≥ 130 mmHg or diastolic ≥ 85 mmHg), (3) low high-density lipoprotein cholesterol level (< 40 mmHg/dL in men and < 50 mm/dL in women), (4) high triglyceride level (≥ 150 mg/dL), and (5) high glucose level (≥ 100 mg/dL).

We controlled for participant’s covariates, such as sociodemographic and socioeconomic factors, health behaviors, and health conditions, in this study. The sociodemographic factors were age (19–29, 30–39, 40–49, 50–59, and ≥ 60 years) and sex (male and female). The socioeconomic factors were education level (middle school or lower, high school, or university or higher), region (metropolitan or rural area), marital status (married or not married), occupation (white collar, pink collar, blue collar, or unemployed), and household income (high, middle-high, middle-low, or low). Health conditions included subjective health condition (good, normal, or bad); stress (yes or no); and family history of hypertension, diabetes mellitus, and/or hyperlipidemia (yes or no).

### Statistical analysis

To confirm the association between lifestyle risk factors and the risk of metabolic syndrome, the covariates were compared using the chi-squared test. Multiple logistic regression analysis was performed to evaluate the relationship between lifestyle risk factors and metabolic syndrome. The results were reported using odds ratios (ORs) and confidence intervals (CIs). Model fitting was performed using the PROC SURVEYLOGISTIC procedure and application of weight procedures, cluster, and strata. The data were analyzed and further stratified by sex by using SAS 9.4 (SAS Institute Inc; Cary, North Carolina). Statistical significance was set at P < 0.05.

### Ethics statement

The KCDA Institutional Review Board (2018-01-03-P-A) approved the protocols of the research, and data release for the seventh KNHANES (2016–2018). All participants provided written informed consent for collecting data before participating in the survey. This study was conducted in accordance to the guidelines of the Declaration of Helsinki-ethnical principles for medical research involving human subjects.

## Results

Table 1 shows the general characteristics of the study subjects. There were 2835 male and 4160 female in this study. People at risk of metabolic syndrome comprised 726 (25.6%) male and 517 (12.4%) female. Participants were also grouped into five categories based on the number of lifestyle factors. In the male group, 189 (6.7%) did not have lifestyle risk factors, 786 (27.7%) had one lifestyle risk factor, 1076 (38.0%) had two lifestyle risk factors, 576 (20.3%) had three lifestyle risk factors, and 208 (7.3%) had four or more lifestyle risk factors. In the female group, 522 (12.5%) did not have lifestyle risk factors, 1733 (41.7%) had one lifestyle risk factor, 1465 (35.2%) had two lifestyle risk factors, 393 (9.4%) had three lifestyle risk factors, and 47 (1.1%) had four or more lifestyle risk factors.

**Table 1:** General characteristics of the study population.

Variables	Metabolic syndrome													
	Male							Female						
	Total		Yes		No		P-value	Total		Yes		No		P-value
	N	%	N	%	N	%		N	%	N	%	N	%	
Total	2835	100	726	25.6	2,109	74.4		4160	100	517	12.4	3,643	87.6	
**The number of poor lifestyle factor**														
0	189	6.7	36	19.0	153	81.0	< 0.0001	522	12.5	47	9.0	475	91.0	< 0.0001
1	786	27.7	177	22.5	609	77.5		1733	41.7	207	11.9	1526	88.1	
2	1076	38.0	276	25.7	800	74.3		1465	35.2	203	13.9	1262	86.1	
3	576	20.3	165	28.6	411	71.4		393	9.4	54	13.7	339	86.3	
≥ 4	208	7.3	72	34.6	136	65.4		47	1.1	6	12.8	41	87.2	
**Age (years)**														
19–29	617	21.8	66	10.7	551	89.3	< 0.0001	705	16.9	22	3.1	683	96.9	< 0.0001
30–39	676	23.8	181	26.8	495	73.2		993	23.9	79	8.0	914	92.0	
40–49	625	22.0	188	30.1	437	69.9		1079	25.9	130	12.0	949	88.0	
50–59	452	15.9	158	35.0	294	65.0		798	19.2	121	15.2	677	84.8	
≥ 60	465	16.4	133	28.6	332	71.4		585	14.1	165	28.2	420	71.8	
**Marital status**														
Living w/ spouse	1821	64.2	557	30.6	1264	69.4	< 0.0001	2922	70.2	384	13.1	2538	86.9	0.032
Living w/o spouse	1014	35.8	169	16.7	845	83.3		1238	29.8	133	10.7	1105	89.3	
**Region**														
Capital area	1865	65.8	437	23.4	1428	76.6	< 0.0001	2756	66.3	337	12.2	2419	87.8	0.584
Rural	970	34.2	289	29.8	681	70.2		1404	33.8	180	12.8	1224	87.2	
**Occupational categories**^**a**^														
White	1101	38.8	300	27.2	801	72.8	0.002	1348	32.4	97	7.2	1251	92.8	< 0.0001
Pink	336	11.9	92	27.4	244	72.6		641	15.4	86	13.4	555	86.6	
Blue	778	27.4	211	27.1	567	72.9		435	10.5	70	16.1	365	83.9	
Inoccupation	620	21.9	123	19.8	497	80.2		1736	41.7	264	15.2	1472	84.8	
**Educational level**														
Middle school or less	295	10.4	84	28.5	211	71.5	0.311	614	14.8	161	26.2	453	73.8	< 0.0001
High school	1042	36.8	253	24.3	789	75.7		1448	34.8	183	12.6	1265	87.4	
College or over	1498	52.8	389	26.0	1109	74.0		2098	50.4	173	8.2	1925	91.8	
**Household income**														
Low	265	9.3	72	27.2	193	72.8	0.743	396	9.5	97	24.5	299	75.5	< 0.0001
Mid-low	594	21.0	156	26.3	438	73.7		925	22.2	129	13.9	796	86.1	
Mid-high	879	31.0	229	26.1	650	73.9		1294	31.1	156	12.1	1138	87.9	
High	1097	38.7	269	24.5	828	75.5		1545	37.1	135	8.7	1410	91.3	
**Perceived stress**														
Yes	769	27.1	205	26.7	564	73.3	0.435	1236	29.7	144	11.7	1092	88.3	0.323
No	2066	72.9	521	25.2	1545	74.8		2924	70.3	373	12.8	2551	87.2	
**Subjective health status**														
Good	1075	37.9	226	21.0	849	79.0	< 0.0001	1391	33.4	123	8.8	1268	91.2	< 0.0001
Normal	1472	51.9	416	28.3	1056	71.7		2206	53.0	301	13.6	1905	86.4	
Bad	288	10.2	84	29.2	204	70.8		563	13.5	93	16.5	470	83.5	
**Family history of HTN**														
No	1874	66.1	471	25.1	1403	74.9	0.418	2446	58.8	310	12.7	2136	87.3	0.566
Yes	961	33.9	255	26.5	706	73.5		1714	41.2	207	12.1	1507	87.9	
**Family history of DM**														
No	2300	81.1	551	24.0	1749	76.0	< 0.0001	3263	78.4	390	12.0	2873	88.0	0.070
Yes	535	18.9	175	32.7	360	67.3		897	21.6	127	14.2	770	85.8	
**Family history of hyperlipidemia**														
No	2656	93.7	684	25.8	1972	74.2	0.497	3798	91.3	484	12.7	3314	87.3	0.030
Yes	179	6.3	42	23.5	137	76.5		362	8.7	33	9.1	329	90.9	
**Lifestyle factor**														
**Sedentary time**														
Normal	1216	42.9	297	24.4	919	75.6	0.211	1962	47.2	245	12.5	1717	87.5	0.913
Less or over	1619	57.1	429	26.5	1190	73.5		2198	52.8	272	12.4	1926	87.6	
**Alcohol use disorder**														
Never or moderate	1875	66.1	438	23.4	1437	76.6	0.000	3833	92.1	478	12.5	3355	87.5	0.7750
Severe	960	33.9	288	30.0	672	70.0		327	7.9	39	11.9	288	88.1	
**Smoking**														
Nonsmoker or Ex-smoker	1832	64.6	436	23.8	1396	76.2	0.003	3944	94.8	488	12.4	3456	87.6	0.6480
Current smoker	1003	35.4	290	28.9	713	71.1		216	5.2	29	13.4	187	86.6	
**Diet intake**														
Normal	1417	50.0	364	25.7	1053	74.3	0.923	1681	40.4	172	10.2	1509	89.8	0.0010
Over	1418	50.0	362	25.5	1056	74.5		2479	59.6	345	13.9	2134	86.1	
**Sleep duration**														
Normal	2317	81.7	578	24.9	1739	75.1	0.003	3349	80.5	403	12.0	2946	88.0	0.1170
Less or over	518	18.3	148	28.6	370	71.4		811	19.5	114	14.1	697	85.9	

Inoccupation group includes housewife.

^a^Three groups (white, pink, blue) based on International Standard Classification Occupations codes.

Table 2 displays the findings of the multiple logistic regression analysis stratified by sex of the association between the number of lifestyle risk factors and the risk of metabolic syndrome. Male with over four lifestyle risk factors had a higher OR for metabolic syndrome risk (over four lifestyle factors: OR 1.97, CI 1.18–3.27).Female with more than one lifestyle risk factor had a higher OR for metabolic syndrome risk (one lifestyle factor: OR 1.58, CI 1.10–2.28; two lifestyle factors: OR 2.08, CI 1.39–3.11; three lifestyle factors: OR 1.94, CI 1.20–3.13).

**Table 2:** Results of factors associated with Metabolic syndrome.

Variables	Metabolic syndrome			
	Male		Female	
	**OR**	95% CI	OR	95% CI
**Total**				
**The number of poor lifestyle factor**				
0	1.00		1.00	
1	0.95	(0.61–1.48)	1.58	(1.10–2.28)
2	1.28	(0.82–1.99)	2.08	(1.39–3.11)
3	1.42	(0.89–2.26)	1.94	(1.20–3.13)
4	1.97	(1.18–3.27)	2.58	(0.93–7.15)
**Age (years)**				
19–29	1.00		1.00	
30–39	2.53	(1.71–3.74)	2.35	(1.22–4.51)
40–49	2.78	(1.85–4.18)	4.06	(2.18–7.55)
50–59	3.57	(2.37–5.37)	4.71	(2.51–8.84)
≥ 60	3.11	(1.96–4.94)	7.72	(4.06–14.67)
**Marital status**				
Living wtih spouse	1.00		1.00	
Living without spouse	0.75	(0.57–1.00)	0.92	(0.66–1.29)
**Region**				
Metropolitan city	1.00		1.00	
Rural	1.43	(1.15–1.77)	1.11	(0.88–1.40)
**Occupational categories**^**a**^				
White	1.00		1.00	
Pink	0.97	(0.71–1.32)	1.53	(1.05–2.22)
Blue	0.81	(0.62–1.06)	1.16	(0.79–1.70)
Inoccupation	0.80	(0.57–1.13)	1.60	(1.21–2.13)
**Educational level**				
Middle school or less	1.00	(0.69–1.44)	1.43	(0.95–2.14)
High school	1.22	(0.94–1.58)	1.13	(0.86–1.49)
College or over	1.00		1.00	
**Household income**				
Low	1.34	(0.89–2.01)	2.00	(1.37–2.93)
Mid-low	1.22	(0.90–1.64)	1.37	(1.01–1.87)
Mid-high	1.18	(0.93–1.49)	1.31	(0.98–1.74)
High	1.00		1.00	
**Subjective health status**				
Good	1.00		1.00	
Normal	1.41	(1.13–1.77)	1.37	(1.04–1.80)
Bad	1.77	(1.25–2.51)	1.44	(1.04–2.01)
**Percieved stress**				
Yes	0.94	(0.75–1.19)	1.01	(0.80–1.27)
No	1.00		1.00	
**Family history of HTN**				
Yes	0.98	(0.80–1.20)	1.09	(0.86–1.37)
No	1.00		1.00	
**Family history of DM**				
Yes	1.54	(1.22–1.95)	1.25	(0.98–1.60)
No	1.00		1.00	
**Family history of Hyperlipidemia**				
Yes	1.06	(0.72–1.58)	0.84	(0.53–1.34)
No	1.00		1.00	

^a^Three groups (white, pink, blue) based on the International Standard Classification Occupations codes.

Inoccupation group includes housewives.

Table 3 shows subgroup analysis findings stratified by dependent variables. Apparently, a higher frequency of poor lifestyle factors was considerably associated with an increased risk of abdominal obesity, hypertension, and elevated triglyceride levels. Especially, triglyceride levels were more likelihood of metabolic syndrome among both male and female. (men: 2 lifestyle factor: OR 1.77, CI 1.18–2.66; 3 lifestyle factors: OR 2.30, CI 1.50–3.52; 4 or more lifestyle factors: OR 3.29, CI 1.99–5.45; women: 2 lifestyle factor: OR 1.98, CI 1.37–2.87; 3 lifestyle factors: OR 2.31, CI 1.50–3.54; 4 or more lifestyle factors: OR 3.62, CI 1.62–8.09).

**Table 3:** The results of subgroup analysis stratified by metabolic syndrome.

Variables	Metabolic syndrome components									
	Abdominal obesity		High BP		Low HDL		High TG		High Glucose	
	OR	95% CI	OR	95% CI	OR	95% CI	OR	95% CI	OR	95% CI
**Male**										
**The number of poor lifestyle factor**										
0	1.00		1.00		1.00		1.00		1.00	
1	1.01	(0.68–1.50)	0.99	(0.66–1.50)	0.72	(0.48–1.08)	1.21	(0.79–1.83)	0.91	(0.61–1.35)
2	1.29	(0.88–1.88)	1.15	(0.78–1.71)	0.72	(0.48–1.09)	1.77	(1.18–2.66)	1.01	(0.68–1.50)
3	1.27	(0.85–1.90)	1.15	(0.76–1.75)	0.77	(0.49–1.19)	2.30	(1.50–3.52)	1.05	(0.68–1.62)
≥ 4	1.60	(0.98–2.62)	1.48	(0.92–2.39)	0.80	(0.47–1.37)	3.29	(1.99–5.45)	0.97	(0.60–1.57)
**Female**										
**The number of poor lifestyle factor**										
0	1.00		1.00		1.00		1.00		1.00	
1	1.10	(0.78–1.55)	1.19	(0.85–1.65)	1.02	(0.81–1.30)	1.91	(1.35–2.69)	1.33	(0.99–1.77)
2	1.51	(1.10–2.07)	1.47	(1.05–2.05)	1.05	(0.82–1.34)	1.98	(1.37–2.87)	1.35	(0.98–1.85)
3	1.53	(1.00–2.33)	1.82	(1.17–2.82)	1.16	(0.86–1.57)	2.31	(1.50–3.54)	1.22	(0.80–1.85)
≥ 4	1.07	(0.36–3.16)	0.64	(0.19–2.16)	1.38	(0.71–2.68)	3.62	(1.62–8.09)	1.91	(0.63–5.79)

*Adjusted for other covariates.

Table 4 displays logistic regression analysis results stratified by variables of poor lifestyle. People who sat longer is more likelihood of metabolic syndrome among both male and female. (male: Q4 sedentary time: OR 1.63, CI 1.21–2.19; female: Q4 sedentary time: OR 1.85, CI 1.31–2.62).

**Table 4:** The results of subgroup analysis stratified by lifestyle factors.

Variables	Metabolic syndrome components			
	Male		Female	
	OR	95% CI	OR	95% CI
**Sedentary time**				
Q1	1.00		1.00	
Q2	1.22	(0.91–1.63)	1.44	(1.03–2.01)
Q3	1.44	(1.10–1.89)	1.82	(1.32–2.52)
Q4	1.63	(1.21–2.19)	1.85	(1.31–2.62)
**Alcohol use disorder**				
Audit < 3	1.00		1.00	
3 ≤ Audit < 8	1.29	(1.00–1.67)	0.74	(0.54–1.00)
Audit ≥ 8	1.66	(1.30–2.13)	1.20	(0.76–1.88)
**Smoking**				
Nonsmoker	1.00		1.00	
Ex-smoker	1.02	(0.78–1.31)	0.91	(0.56–1.47)
Smoker	1.14	(0.87–1.49)	0.95	(0.56–1.61)
**Diet intake**				
Normal	1.00		1.00	
Over carbohydrate	0.97	(0.78–1.21)	1.24	(0.97–1.58)
Over fat	0.94	(0.70–1.25)	1.14	(0.78–1.67)
**Sleep duration**				
Less	0.85	(0.63–1.15)	0.80	(0.59–1.09)
Normal	1.00		1.00	
Over	0.96	(0.61–1.53)	0.71	(0.43–1.17)

*Adjusted for other covariates.

## Discussion

In this study, we found that people who have more poor lifestyle behaviors tended to have an increased risk of metabolic syndrome in the Korean adult population. And the association with metabolic syndrome was more shown in female group than male group. In prior studies, sex difference in lifestyle factor were shown the relationship with metabolic syndrome^21–23^. This sex difference was derived from energy metabolism and difference of physical characteristic. Women have higher proportion of body fat than men and have different effects on hormones^24^.

In our study, women who had four poor lifestyle factors did not show any significant association because of poor data regarding smoking status. Our study was based on self-reported data. According to some previous studies, validity assessments of self-reported smoking status data among women have revealed discrepancies in self-reported, non-smoker data^25^. So we think that the value with over four lifestyle risk factors in female is affected to the defect.

The prevalence and trend of metabolic syndrome in Korea vary widely, that is, 5.2–35.3% in the male population and 9.0–39.2% in the female population^26^. In our study, the prevalence of metabolic syndrome was 25.6% and 12.4% in the male and female groups, respectively. We excluded patients with diagnoses of angina, myocardial infarction, and stroke because baseline cardiovascular diseases and stroke have a strong relationship with the risk of metabolic syndrome and the diagnoses of disease is a great trigger to change people’s lifestyle. Further, people undergoing treatment for hypertension, diabetes mellitus, and hyperlipidemia have been linked to lifestyle behaviors in previous studies, and if we were to include that subgroup in our study, the association between lifestyle and metabolic syndrome would have been underestimated.

People with a greater number of poor lifestyle factors tended to be increased likelihood of abdominal obesity, hypertension and TG. level. Some studies have investigated the combination of metabolic syndrome components^23,27,28^. For abdominally obese Korean females and males, the most prevalent metabolic syndrome combination is “triglyceride + HDL-cholesterol” and “triglyceride + blood pressure”, respectively^27^. In the other studies, the combination of abdominal obesity, low HDL and hypertriglyceridemia is significantly different between socioeconomic status and sex in Korea^23^. The combination is more prevalence in lower SES group and female. In other country, the relationship between abdominal obesity, high TG and reverse HDL-C is observed^28^. Therefore, components of metabolic syndrome may have different prevalence with regard to external effect.

Several studies have demonstrated that long sedentary time has a negative effect on health outcomes^29^. Sedentary behavior, generally characterized by a mere lack of physical activity, has also been associated with worse health outcomes, such as obesity, diabetes, insulin resistance, metabolic syndrome, and cardiovascular disease^30,31^. Some studies have also found alcohol consumption to have a positive relationship with metabolic syndrome. Alcohol intake has been positively associated with body weight, high-density lipoprotein cholesterol levels, and hypertension^32^. Current smoking was found to affect all components of metabolic syndrome in the male group only. In this study, the proportion of current smokers was 38.6% and 7% in the male and female groups, respectively. Smoking is known to increase insulin resistance and affect lipid metabolism in the body^33^. Smoking has also been considered to influence adverse abdominal obesity^34^, and those who quit smoking may exhibit high levels of hyperglycemia and triglycerides^35^. But in our study, it was not significant, which is that smoking surveyed by self- report is unreliable as mentioned above.

For more effective lifestyle interventions targeting metabolic syndrome, the current study proposes the following recommendations. The initial management of metabolic syndrome entails lifestyle modifications recommended by The National Cholesterol Education Panel Adult Treatment Panel^36^. First, when formulating a preventive program against metabolic syndrome in adults, we should consider that the effectiveness of the program potentially varies by sex. Second, according to sex, programs should target specific lifestyle risk factors for intervention. When evaluating people’s risks of metabolic syndrome, the number of poor lifestyle behaviors should be taken into account. Third, further research is required to establish reasons underlying sex differences in lifestyle behaviors that affect the risk of metabolic syndrome. Further, an investigation of the associations between the various components of metabolic syndrome and each lifestyle behavior is imperative.

The current study has certain limitations. First, this study was based on data from a cross-sectional study. Therefore, whereas associations could be confirmed, causality could not be evaluated. Second, our study relied on self-reported data. Hence, the measurement of lifestyle risk factors might not have been accurate. Future studies will need to perform precise measurements of lifestyle risk factors. Third, as the cutoff points for lifestyle risk factors were adopted from the KNHANES, it may be difficult to generalize our findings to different settings or populations^12^. Despite these limitations, this study has several strengths. We used the most recent, nationally representative database to determine the association between lifestyle risk factors and the risk of metabolic syndrome. Therefore, the results obtained are highly representative of healthy South Korean adults. In addition, we utilized American Heart Association/National Heart, Lung, and Blood Institutes Scientific Statement criteria for defining metabolic syndrome in Asians and diagnosing patients with metabolic syndrome.

## Conclusion

This study found a significant association between the number of lifestyle risk factors and the risk of metabolic syndrome in Korean adults. The greater the number of lifestyle risk factors, the higher the risk of metabolic syndrome in both sexes. People with a greater number of poor lifestyle behaviors tended to exhibit increased likelihood of abdominal obesity, hypertension, and especially elevated triglyceride levels. Sedentary time was also strongly associated with the risk of metabolic syndrome.

## Data Availability

The datasets analyzed during the current study are available the KNHANES official website (https://knhanes.kdca.go.kr/knhanes/main.do).
